# Shared Virtual Reality Experiences during the COVID-19 Pandemic: Exploring the Gratifications and Effects of Engagement with Immersive Videos

**DOI:** 10.3390/ijerph19095056

**Published:** 2022-04-21

**Authors:** Yang Cheng, Yuan Wang, Wen Zhao

**Affiliations:** 1 Department of Communication, North Carolina State University, Raleigh, NC 27695, USA; 2 Department of Media and Communication, City University of Hong Kong, Hong Kong, China; yuan.wang@cityu.edu.hk; 3 Department of Communication, Fairfield University, Fairfield, CT 06824, USA; wzhao@fairfield.edu

**Keywords:** COVID-19, engagement, global recovery, gratifications, immersive videos, virtual reality

## Abstract

The coronavirus (COVID-19) pandemic and recent economic recession have been impacting many people’s mental health. The experience of social distancing created new hardships for people who already reported symptoms of depression or anxiety. In these circumstances, new technologies, such as immersive virtual reality (VR) videos, could serve as useful tools for facilitating interactions, emotional sharing, and information processing within a virtual environment. In this study, researchers aimed to enrich the information processing literature by focusing on the uses and gratifications of 360-degree VR videos during the pandemic. Through employing survey research with 1422 participants located in the U.S. and structural equation modeling for data analysis, this study found that five types of gratification, including utilitarian (i.e., navigation), hedonic (i.e., enjoyment), sensual (i.e., realism), social (i.e., community), and symbolic (i.e., coolness), significantly motivated users to use such immersive videos. Simultaneously, data demonstrated that these five types of gratification could influence users’ cognitive engagement with virtual content. In addition, such VR engagement facilitated users’ positive attitudes toward immersive videos and continued usage of them. The findings provided practical implications for COVID-19 global recovery as well.

## 1. Introduction

When billions were made to isolate individuals at home during the coronavirus (COVID-19) pandemic, it gave rise to new technologies, such as virtual reality (VR). VR, defined as a communication platform that enables audiences to interact with a computer-generated, three-dimensional virtual environment [1], has helped numerous people to get through their challenging quarantine lives. For instance, Baker et al. [2] found that VR not only helped individuals engage with their families via virtual travel, but also created shared experiences with family members in real life. Meanwhile, VR spurred an economic recovery during the post-COVID-19 period [3]. Shaped by the COVID-19 crisis, the global VR market was expected to surge rapidly from $6.30 billion in 2021 to $84.09 billion in 2028 [4]. In 2021, more than 171 million users worldwide had adopted VR, 58.9 million Americans used it more than once per month in 2021, and the number of active users will reach 65.9 million in 2023 [5].

With the development of VR and 360-degree platforms, more and more organizations have started incorporating VR-enabled 360-degree technology in their health promotion, marketing, and crisis communication campaigns to create engaging information to connect with their stakeholders [6,7,8]. For example, the British Broadcasting Corporation (BBC) recently designed a 360-degree VR video to raise awareness about mental health issues [9]. In this video, the audience could directly experience the main character’s first-person perspective (the character had anxiety issues) by dragging the mouse around to get the desired angle [9].

Researchers found that 360-degree video, as VR-powered production technology, could enrich immersive storytelling [10], increase transportation in the mediated environment [11], create visual realism [12], enhance audience engagement [8], and ultimately generate persuasive outcomes [6]. Engagement here refers to “a mental state that is accompanied by active and sustained, even complex, cognitive processing” [13] (p. 923). In the digitally mediated communication context, engagement has a significant influence on users’ decision making [7], since such engagement with mediated environments can provide audiences with opportunities to negotiate the meanings of media information and create dialogues through new forms of immersive media content [14]. As engagement is connected to these persuasive effects, scholars have begun to explore the factors that affect individuals’ engagement with the immersive media content, including users’ authentic VR experiences [15] and individual personalities, such as an immersive tendency [8]. However, the antecedents or outcomes of VR engagement were not fully investigated within the context of virtual content during crises before our study. Which gratifications might motivate users to engage in VR videos remained unknown till now. Few studies have ever focused on the shared VR experiences during the pandemic and discussed the global recovery via VR videos.

To address these research gaps, we built upon previous literature [16,17] and applied uses and gratifications (U&G) theory as a theoretical explanation to examine five types of gratification sought (i.e., utilitarian, hedonic, sensual, social, and symbolic gratifications) with the 360-degree VR video. Data were collected through a cross-sectional survey of 1422 nationally representative participants in the United States.

**Figure 1:**
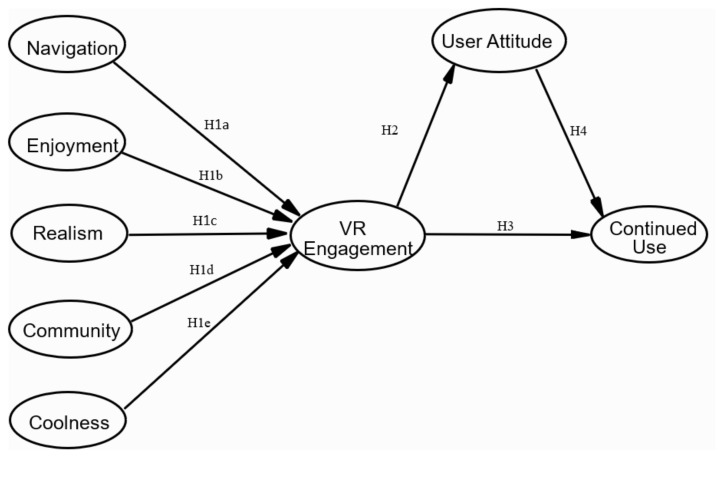
The conceptual model.

The purposes of this study were threefold. First, we aimed to enhance the U&G approach in virtual communication by examining how sought gratifications are associated with users’ engagement with 360-degree immersive videos. Second, we intended to shed light on the information processing during the COVID-19 pandemic by addressing the strategic use of the 360-degree immersive videos and promulgating that users’ engagement with such immersive videos might assist users in forming positive attitudes toward them and continued use (as shown in Figure 1). Finally, the findings of this research also provide practical implications for global recovery from the pandemic and offer suggestions for VR businesses, practitioners, and individuals within the crisis.

## 2. Literature Review

### 2.1. Uses and Gratifications Theory

U&G theory has been one of the most applied theories in communication research. It can explain how individuals use media to fulfill their needs, the motivations for their media usage, and the functions or outcomes resulting from needs, motivations, and behavior [18]. Individuals’ needs are divided into five categories: cognitive, social integrative, affective, personal integrative, and tension-release needs [19]. Early U&G studies were mainly descriptive and focused on identifying motivations of media usage, whereas later research was more systematic and examined the effects of media usage [20]. The U&G approach has been widely applied to various media platforms in communication studies, such as newspapers [21], television [22], the Internet [23], and social media [24].

More recently, building upon Katz et al.’s classification [18], Rauschnabel [16] proposed a theoretical framework focusing on utilitarian (i.e., cognitive need), hedonic (i.e., tension-release need), sensual (i.e., affective need), social (i.e., social integrative need), and symbolic (i.e., personal integrative need) gratifications and applied this framework to augmented reality (AR) smart glasses. Given that AR and VR share similar operational modes and development principles [25], a couple of researchers began to use the U&G approach to study the usage and gratifications of VR technology. For example, Kim and Lee [26] adopted the U&G approach to examine how and why individuals experience 360-degree VR art and found that pursuing learning from entertainment is immersive. According to Pantano and Corvello [27], when travelers derive enjoyment from their VR experiences, their decisions are affected by their VR tours. Drawing from Rauschnabel’s [16] categorization of gratifications and previous literature on VR gratifications (see a summary in Table 1), we adopted the utilitarian, hedonic, sensual, social, and symbolic gratifications as five dimensions to explore users’ motivations of using VR videos. Relevant literature on these gratifications and their associations with VR engagement are presented below.

### 2.2. VR Engagement

Before the full immersion of digital technologies, scholars used the term “bricolage” to describe the experience of engagement with an interactive mediated platform, such as internet surfing [34]. Shih [34] emphasized tinkering and manipulation as two important aspects of navigating and learning product information in immersive media environments. Tinkering refers interacting with virtual objects (e.g., web surfers) while the program processes the information in the immediate environment and surroundings. Manipulation refers to controlling the media information. Shih [34] (p. 660) labeled this active tinkering and manipulating in cyberspace “bricolage,” which is the “tinkering and manipulation of objects around one’s immediate environment to develop and assimilate ideas.” Following this definition, researchers conceptualized engagement as meaningful interactions that involve cognitive processing, problem solving, and making sense of narratives [35,36].

Furthermore, another definition of engagement is “turning on a prospect to a brand idea enhanced by the surrounding context” [37] (p. 355). This working conceptualization considered the engagement process as the individuals’ interactive experiences with a brand. As Wang [37] argued, engagement plays a crucial role in processing information, since people are motivated to engage with the brand information in media and the immersive environment surrounding them. For example, active media users could use diverse digital platforms and features which would allow them to access online content [38]. As Mollen and Wilson [13] argued, online engagement can be the active interactions between individuals and brands, requiring persistent cognitive processing of the narratives and brand values. In the digital learning environment, Herrington et al. [39] emphasized this cognitive aspect of the engagement and argued that digital engagement happens when users could give themselves up to a representative action. From the dialogic perspective, both VR content creators and viewers are engaged in dialogue creations within the communication process [12]. Thus, VR experiences could enhance users’ emotional attachment to a brand [40] and generate robust narrative engagement [41].

In the context of VR films, Chen and Wang [40] discovered that participation with product placement in the VR environment reflected the allocation of cognitive resources and the co-creation of meaningful experiences. According to Gruenewald and Witteborn [42], VR engagement could also create a personalized experience for users in a computer-simulated virtual world that would allow them to engage with global issues. Specifically, such immersive humanitarian VR videos could satisfy the needs of users through the details of the virtual environment and create engagement with people who have suffered in a refugee camp or rural Afghanistan [42]. Grounded in the prior research above, this current study conceptualized VR engagement as an interactive experience where users actively engage in cognitive information processing and develop meaningful personalized connections in a VR video-mediated environment.

### 2.3. Gratifications and VR Engagement

#### 2.3.1. Utilitarian Gratification

A few researchers have explored the relationship between utilitarian gratification (i.e., navigation) and VR engagement [28]. Utilitarian gratification is conceptualized as “the gratification related to enhanced effectiveness and efficiency of social activities” [43] (p. 211). As a specific type of utilitarian gratification, navigability is defined as “the affordance that allows user movement through the medium” [44] (p. 516). Information science scholars have used utilitarian constructs to differentiate information systems, rationalize user experience concepts, and study the outcomes of technology usage [45,46,47]. According to Alzayat and Lee [28], customers who interacted with products in VR were engaged in their shopping experiences. Similarly, we assume that individuals who access effective visual aids and interfaces provided by 360-degree VR videos are more likely to think about what is presented in such videos. Thus, we posited the following hypothesis.

**H1a.**  *Using 360-degree VR videos for navigation has a positive effect on VR engagement*.

#### 2.3.2. Hedonic Gratification

Hedonic gratification refers to “the pleasure, relaxation, self-determination, and fulfillment of socio-psychological needs” due to the use of communication technology [43] (p. 211). Hedonic gratification affects individuals’ decisions on technology usage [48]. According to Sledgianowski and Kulviwat [49], individuals use social media mainly to satisfy their hedonic needs. In particular, this study focuses on enjoyment as a hedonic gratification based on the U&G framework [20]. Enjoyment refers to “the idea of distracting oneself from everyday activities by consuming entertaining media” [16] (p. 561).

Enjoyment gratification is a major reason for individuals to use VR technology, such as VR tourism [50]. According to Kim and Hall [33], tourists have engaged in VR tourism activities for enjoyment gratification. Furthermore, tourists’ enjoyment desire leads to the intention to visit travel destinations in VR [51]. Thus, we argue that individuals who consume a VR-powered 360-degree video for enjoyment tend to imagine the world presented in that video. Therefore, the following hypothesis was posed.

**H1b.**  *Using 360-degree VR videos for enjoyment has a positive effect on VR engagement*.

#### 2.3.3. Sensual Gratification

Sensual gratification refers to the “benefits derived from the stimulation of various human senses” [16] (p. 561). This type of gratification has been used to explain audiences’ media (e.g., television and the Internet) selection and consumption behavior [26]. For instance, it can explain why audiences may consume specific media content [52]. According to Lin and Tsai [53], a user with higher sensual gratification is more likely to depend on the Internet. Kim and Lee [26] examined the motivations for users viewing 360-degree VR art and found that their sensation-seeking tendency influenced their motivation for viewing VR art, including learning from entertainment. Given that realism is a type of sensual gratification, we assume that if a person integrates a VR video realistically into his/her perception of the real world [54], he/she is more likely to consider whether the presentation in that video has personal meaning for himself/herself. Thus, the following hypothesis was proposed.

**H1c.**  *Using 360-degree VR videos for realism has a positive effect on VR engagement*.

#### 2.3.4. Social Gratification

Social gratification refers to social presence and social interaction [5]. Researchers have connected this gratification to digital media technologies, such as social media [55], AR-smart glasses [16], and VR [56]. For example, AR users may connect and form online communities to share their experiences [16]. Gruenewald and Witteborn [42] indicated that social gratification could explain users’ motivations for watching VR movies. VR game users’ social gratification influenced their psychological dependency on such games [56] and led to an interactive, engaging experience. Similarly, we argue that users of VR videos who connect with other users tend to perceive an interactive experience where they establish meaningful connections with the content. Accordingly, the following hypothesis was posited.

**H1d.**  *Using 360-degree VR videos for social community has a positive effect on VR engagement*.

#### 2.3.5. Symbolic Gratification

Symbolic gratification covers coolness, self-expression, promoting oneself, and position [16]. It could be assessed based on personal integrative needs [16]. Audiences’ media consumption may hint at symbolic behavior that expresses them externally and recognizes their personalities [57,58]. According to Kim and Lee [26], audiences’ symbolic gratification (i.e., self-expression) influences their motivations for viewing 360-degree VR art. Given that coolness is an important sub-category of symbolic gratification [16], we assumed that if audiences consume VR videos to fulfill their coolness gratification, they are more likely to engage with such videos. Thus, the hypothesis below was produced.

**H1e.**  *Using 360-degree VR videos for coolness has a positive effect on VR engagement*.

### 2.4. VR Engagement and User Attitude

Attitudes, positive or negative feelings toward certain subjects, reflect an individuals’ standards of good and bad, right and wrong, etc. [59]. In the literature on VR communication, users’ attitudes toward VR-powered videos have been explored as a dimension of users’ evaluations [8]. User attitudes toward the VR video refer to “outcomes of perceived congruence of self with the message or cognitive evaluations” [8] (p. 7).

As engagement represents the cognitive processing of information via VR videos, it is expected that the higher the levels of engagement users develop, the more likely are stronger desires to process the information and subsequently generate positive responses toward the VR videos. As VR and 360-degree films provide media richness and greater user control over the media contents, such immersive VR features can increase the presence of feelings in the computer-mediated environment and lead to more favorable persuasion outcomes [60]. Further, prior research has found that immersive media such as VR and 360-degree films could positively affect attitude formation [7]. Hence, we proposed the following hypothesis:

**H2.**  *Users’ engagement with the 360-degree VR video has a positive effect on their attitudes toward it*.

### 2.5. VR Engagement and Continued Use

We focused on users’ subjective intentions to continue using VR-assisted immersive videos within this study. These intentions are defined as users’ intentions to use certain products or services continuously, an important concept that has attracted heated discussions. Particularly concerning VR engagement and continued use in the context of immersive VR communication, very few scholars have explored the effects of VR engagement on users’ behaviors, such as continued use. As Mollen and Wilson [13] (p. 923) argued that the construct of online engagement plays a vital role in explaining optimal consumer behavioral outcomes, since the engagement process can help consumers find “utility and emotional congruence with the ‘whole’ of the educational message or narrative or brand.” Likewise, in Markowitz and Bailenson’s [61] recent paper on VR and climate issues, they pointed out that individuals would be more likely to change attitudes and behaviors when the immersive media was more interactive and more personally connected. In line with the above literature, we assumed that VR engagement would positively affect the continued use of 360-degree VR videos.

**H3.**  *VR engagement with the 360-degree VR video has a positive effect on users’ continued use intentions*.

### 2.6. User Attitude and Continued Use

Scholars in the past have identified antecedents of continued use, such as the user’s attitude toward the field of communication in general and new media in particular [62,63,64]. For instance, studies found that positive attitudes strongly affected their intentions to continue using the applications [65,66,67]. Pertami and Sukaatmadja [68], in their research, stated that if a person had positive feelings toward TikTok, they would like to continue using this social media tool. Kosa et al. [69] indicated that players’ positive attitudes toward VR gaming would influence their intentions to play those games. Therefore, we developed the following hypothesis to examine the relationship between user attitude and continuous usage of immersive videos.

**H4.**  *Users’ attitudes toward the 360-degree VR video positively influence continued use intentions*.

## 3. Method

### 3.1. Data Enrollment

After obtaining approval from the Institutional Review Board (IRB), we collected data and enrolled respondents located in the U.S. via Qualtrics, a third-party survey platform. We used stratified and quota random sampling strategies to obtain a representative sample based on percentages of demographic variables from the U.S. census data and sent anonymous links randomly to all participants in September 2020. A total of 4940 participants volunteered to join this study, and 1422 of them fully completed all questions and were ultimately included in the data analysis process.

To ensure the quality of this study, we executed the following two steps. First, we launched a pretest with the first 100 participants; they were asked specific qualitative questions about their gratifications achieved when using 360-degree immersive videos. Then, based on the feedback gathered from the pretest, researchers finalized all the survey questions. At the start of the questionnaire, respondents were given a concise introduction to 360-degree immersive videos. The survey also provided an example of 360-degree immersive video, which used VR technology to empower the public to be informed about mental health problems during the COVID-19 pandemic. Participants were required to watch this 360-degree immersive video for at least five minutes before they could move forward to the following questions on gratifications sought when using immersive videos, their engagement with and attitudes toward such videos, and continued use intentions. Second, during the data collection, we adopted a filter question to ensure all participants had experiences with 360-degree VR videos before joining this study, and we also applied attention-check questions, which included, “If you are showing attention, kindly please choose ‘strongly agree’ to pass.” A total of 3518 participants who neither passed the quality control questions nor completed the questionnaire were excluded from the sample.

### 3.2. Characteristics of Respondents

Among the 1422 participants, 50.1% of them were male (n = 712), whereas 49.9% were female (n = 710). We discovered that 269 respondents (19%) were between 18 and 35; 540 were 36–50 (38%); 336 were 51–65 (23.6%); and 277 were aged 66 and above (19.4%). Regarding ethnicity, 1098 participants (77.2%) were Caucasian/White, 124 were Black or African American (8.7%), 92 were Latino/Hispanic (6.5%), 79 were Asian (5.6%), 15 were American Indian (1.1%), and 14 considered their race as “other” (0.9%). In terms of education, 886 respondents were shown to hold a bachelor’s degree or higher (62.3%). In regard to income, we found that 477 participants (33.5%) had annual household earnings of $100,001 and higher (USD), followed by 242 (17%) who earned between $40,001 and $60,000, 228 (16%) who earned between $20,001 and $40,000, 184 (13%) who earned between $60,001 and $80,000, 146 (10.3%) who earned $20,001 and under, and 145 (10.2%) who earned between $80,001 and $100,000.

### 3.3. Measures

This study adopted a five-point Likert-type scale for all survey questions/statements, ranging from 1 (“strongly disagree”) to 5 (“strongly agree”). In the pretest, researchers checked the reliability and validity of each measurement item. Cronbach’s alpha for the measure of each variable ranged from 0.87 to 0.95. As a result, we kept the original measures and continued with the main study.

#### 3.3.1. Gratifications Sought

Utilitarian gratification (i.e., navigation) was gauged by using items from Wang et al. [17]. Specifically, based on the user’s most recent experience with a 360-degree VR video, he/she was asked to respond to statements such as, “It allows me to link to other pieces of information”; “It offers a number of visual aids for more effective use”; and “The interface helps me every step of the way.” Hedonic gratification (i.e., enjoyment) was measured by using three items from Rathnayake and Winter [70]: “It is fun to explore”; “It lets me play”; and “I enjoy escaping into a virtual world.” To measure sensual gratification (i.e., realism), we adopted three items from Wang et al. [17]. Sample statements were “I know the content is real and not made up”; “It is like communicating face-to-face”; and “The VR experience is very much like real life”. For the measurement of social gratification (i.e., community), we chose three items from Wang et al. [17]: “I can connect with other followers”; “It allows me to expand my social network”; and “It makes me realize that I am part of a community.” Finally, we applied three statements from Rathnayake and Winter’s [70] scale to capture symbolic gratification (i.e., coolness): “It is unique”; “It is innovative”; and “It is different”. We also conducted reliability tests and generated the Cronbach’s alpha value for each of these variables: navigation (α = 0.90), enjoyment (α = 0.90), realism (α = 0.88), community (α = 0.93), and coolness (α = 0.87).

#### 3.3.2. VR Engagement

Based on Von der Pütten et al.’s scale [71], we measured users’ engagement with 360-degree immersive videos using the following three questions: “I thought most about things having to do with the 360-degree VR video”; “I imagined precisely what it must be like to further explore the world presented in the 360-degree VR video”; and “I kept wondering whether the presentation in the 360-degree VR video could have personal meaning for me.” The Cronbach’s alpha of VR engagement was 0.92.

#### 3.3.3. User Attitude

Following previous literature on attitudinal evaluation [72], researchers adopted three items to measure users’ attitudes toward immersive videos. Statements included “The 360-degree VR video is appealing to me,” “[…] attractive to me,” and “[…] interesting to me.” The Cronbach’s alpha value was 0.93.

#### 3.3.4. Continued Use

To measure participants’ intentions of continuing to use immersive videos, this study applied Cheng and Jiang’s [62] scale. It included three questions: “I would like to recommend others to watch 360-degree VR videos”; “I will increase my use of 360-degree VR videos”; and “Continuing to use 360-degree VR videos is something I would do in the future.” The Cronbach’s alpha value was 0.93.

#### 3.3.5. Control Variables

Past studies have indicated that demographic variables (e.g., gender and age) of users and the frequency of using 360-degree VR videos might influence users’ attitudes and intentions to continue using such media [15,62]. We thus measured these variables and controlled them in the final data analysis.

## 4. Findings

### 4.1. Descriptive Statistics

In the Likert scale, options were “low” (1.00–1.99), “moderately low” (2.00–2.99), “neutral” (3), “moderately high” (3.01–3.99), and “high” (4.00–5.00). The scale allows the presentation of descriptive data, such as the mean and standard deviation of each variable. According to the data, respondents generally reported “moderately high” regarding their perceived five types of gratification sought from the 360-degree VR video, which included navigation (M = 3.63, SD = 1.05), enjoyment (M = 3.87, SD = 1.04), realism (M = 3.71, SD = 1.08), community (M = 3.54, SD = 1.16), and coolness (M = 3.96, SD = 0.94). Simultaneously, the data demonstrate moderately high levels of VR engagement (M = 3.80, SD = 1.08), positive attitude toward immersive videos (M = 3.90, SD = 1.11), and positive attitude toward continued use (M = 3.79, SD = 1.12). As shown in Table 2, all examined variables were significantly associated with each other, and the coefficient values were between 0.62 and 0.81.

### 4.2. CFA Results

Based on Hu and Bentler’s [73] joint model testing criterion, either comparative fit index (CFI) ≥ 0.96 and standardized root mean square residual (SRMR) ≤ 0.10 or root mean square error of approximation (RMSEA) ≤ 0.06 and SRMR ≤ 0.10, the CFA model obtained a good fit (χ^2^ = 809.755, df = 214, χ^2^/df = 3.784, CFI = 0.98, TLI = 0.98, NFI = 0.98, SRMR = 0.02, RMSEA = 0.044 (90% CI = 0.041–0.048), n = 1422). As shown in Table 3, factor loadings of all survey questions reached an acceptable range (0.77 to 0.95). Composite reliability (CR) (ranged from 0.85 to 0.95) and the average variance extracted (AVE) (ranged from 0.66 to 0.87) of the measures were calculated as well, confirming that these measurements were valid and reliable. Fornell and Larcker’s [74] method was also adopted to check the discriminant validity of the measurement model. Data from Table 2 found that the square root of each AVE value of the latent variable (0.81–0.93) is higher than the correlation between any other latent variables. Thus, discriminant validity has been supported.

### 4.3. Hypothesis Testing

**Figure 2:**
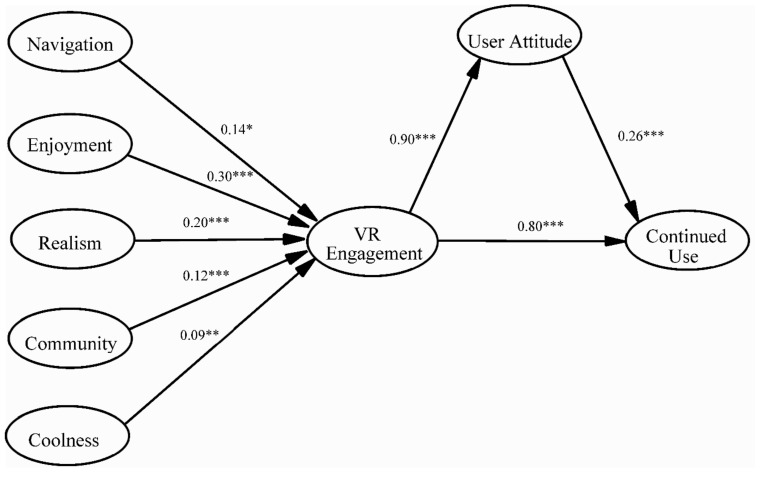
The results of the structural equation model. Note: *** *p* < 0.001; ** *p* < 0.01; * *p* < 0.05.

To test the proposed hypotheses, we performed a structural analysis, and the results indicated that the structural model achieved a satisfactory model fit: χ^2^ = 918.891, df = 253, χ^2^/df = 3.632, CFI = 0.98, TLI = 0.98, NFI = 0.97, SRMR = 0.02, RMSEA = 0.043 (90% CI = 0.040–0.046), n = 1422. As shown in Figure 2, data support the first set of hypotheses (H1a–d) as well, and demonstrate that the five types of gratification sought, navigation (β = 0.14, *p* < 0.05), enjoyment (β = 0.30, *p* < 0.001), realism (β = 0.20, *p* < 0.001), community (β = 0.12, *p* < 0.001), and coolness (β = 0.09, *p* < 0.01), were significantly related to VR engagement. In other words, the higher the level of utilitarian (i.e., navigation), hedonic (i.e., enjoyment), sensual (i.e., realism), social (i.e., community), or symbolic gratification (i.e., coolness) that users sought from immersive videos, the more likely they would cognitively engage in thinking and learning from such videos.

In addition, H2 was supported when our findings reflected that VR engagement could positively affect users’ attitudinal evaluations of the VR video (β = 0.90, *p* < 0.001). Specifically, the higher the level of cognitive thinking invested in immersive VR videos, the higher the likelihood that users would find such videos appealing, interesting, or attractive.

Meanwhile, the data are consistent with H3′s suggestion of VR engagement having a positive influence on continued use intentions (β = 0.80, *p* < 0.001). Lastly, our findings support H4 by displaying the relationship between user attitude and intentions of continued use: user attitude significantly impacted the latter (β = 0.26, *p* < 0.001).

### 4.4. Indirect Effects

To test indirect relationships between the current variables in the structural model, we applied Amos 20 and followed its bias-corrected (BC) bootstrapping procedure (n = 5000 samples). The data first demonstrated that users’ attitudes toward VR videos significantly mediated the association between VR engagement and continued use (β = 0.24, *p* < 0.001, BC 95% CI= [0.14, 0.34]). Furthermore, the findings highlighted the important mediating roles of VR engagement on enjoyment and user attitude (β = 0.27, *p* < 0.001, BC 95% CI= [0.14, 0.42]); community and user attitude (β = 0.11, *p* < 0.05, BC 95% CI= [0.02, 0.20]); and realism and user attitude (β = 0.18, *p* < 0.001, BC 95% CI= [0.08, 0.29]). In addition, indirect effects between gratifications sought and continued use existed: enjoyment and continued use (β = 0.31, *p* < 0.001, BC 95% CI= [0.16, 0.48]); community and continued use (β = 0.13, *p* < 0.05, BC 95% CI= [0.03, 0.23]); and realism and continued use (β = 0.21, *p* < 0.001, BC 95% CI= [0.09, 0.34]).

## 5. Discussion

This study focused on users’ shared experience with 360-degree VR videos during the COVID-19 pandemic. We surveyed 1422 participants located in the United States and analyzed data through structural equation modeling. We found that five types of gratification sought, utilitarian (i.e., navigation), hedonic (i.e., enjoyment), sensual (i.e., realism), social (i.e., community), and symbolic (i.e., coolness), could positively motivate users to engage with 360-degree VR videos. VR engagement maintained positive relationships with users’ attitudes toward and continued intentions to use such VR videos. The attitudes of users further exerted a positive impact on continued use intentions. Theoretical implications and practical suggestions for global recovery are discussed below.

### 5.1. Theoretical Implications

We first enriched the U&G theoretical approach by investigating the five types of gratification sought from VR videos and their relationships with users’ VR engagement during the pandemic. In previous studies, communication scholars intensively examined different types of traditional and social media and developed different categories of gratification based on each type of media [23,24]. For instance, Stafford et al. [75] studied the three kinds of user gratification received from the Internet: process, content, and social motivations. Gan and Li [76] focused on users’ gratification from WeChat, a smartphone application, and proposed four types, hedonic, social, utilitarian, and technology, in their theoretical framework. Cheng et al. [77] identified that people used social media tools on mobile devices during crises because of technological convenience, cognition and recognition needs, affection, entertainment, and fashion/status.

In recent years, some scholars [26,27] found that people were motivated to experience VR art or tourism because of entertainment needs. Other studies supported the social function of VR tools, helping users share their experiences and establish social connections [42]; researchers also investigated the navigability of 360-degree immersive videos [78]. However, the research attention on the gratification of watching 360-degree VR videos was still limited. A comprehensive theoretical framework on varieties of gratification sought from using 360-degree VR videos within the post-pandemic context is lacking. Results of this study filled the research gap and advanced previous literature by integrating five main categories of gratification sought, utilitarian, hedonic, sensual, social, and symbolic, in one theoretical model, and confirmed their significant correlations with VR engagement as well. Interestingly, this study identified sensual gratification (i.e., realism) as a unique motivation for using VR videos that could facilitate users’ engagement. Specifically, if people find immersive videos realistic and like face-to-face communication, then they will likely have a high level of engagement with the virtual content. This study also confirmed that the perceived hedonic (i.e., enjoyment) gratification could serve as a critical precursor to VR engagement. This finding offers additional empirical evidence regarding hedonic gratification and its impact in the VR technology context, supplementing previous literature on the effects of enjoyment in the VR tourism industry [51].

Second, the findings of this research shed light on information processing during the COVID-19 pandemic within the context of VR communication. In the VR-mediated communication environment, engagement closely relates to the persuasive effects; scholars have explored how factors such as users’ authentic VR experiences or immersive tendencies [8,15] might affect their engagement with the immersive media content. However, few studies ever explored both antecedents and outcomes of VR engagement in the context of crises. This study filled the gap by fully supporting the positive influence of gratifications on VR engagement during the pandemic; we also found strong associations between VR engagement, user attitude, and continued use. Specifically, we found that users’ VR engagement largely influenced their positive attitudes toward such tools and their intentions to continue to use these VR videos, broadening prior studies on the outcomes of VR engagement [61].

The results demonstrate the predominant mediating role of VR engagement between the three types of gratification sought (i.e., enjoyment, community, and realism) and user attitudes; and between these three above-mentioned types (i.e., enjoyment, community, and realism) and continued use. Specifically, for users with a low level of engagement with virtual content, the perceived enjoyment, community, and realism from VR videos are less likely to influence their continued use and a positive attitude toward VR videos. However, this research also provides initial evidence that when VR videos emphasize entertainment, social communities, and immersion, they could have a positive effect on users’ attitudes and intentions toward continued use if people display a high level of cognitive information processing of virtual content. These findings further highlight how user attitude or continued use could operate differently between people with different levels of cognitive engagement with virtual content, which extends previous literature on user experience in different digital settings [79,80].

### 5.2. Implications for Global Recovery

As the world is starting to recover from the COVID-19 pandemic, it is necessary to review and examine the shared VR experience and summarize what we could do to advance the recovery of global well-being using such virtual technology. The findings of this study indicate that users were motivated to use 360-degree VR videos because of five major types of motivation (i.e., utilitarian, hedonic, sensual, social, and symbolic gratifications), which further facilitated users’ cognitive information processing in relation to these VR videos. To recover from the pandemic, it is imperative for VR businesses and practitioners to concentrate more on users’ psychological motivations. For instance, in the tourism industry, practitioners could design and offer updated information to increase the coolness and enjoyment of using VR for remote travel. It is beneficial for the reignition of the tourism industry to create and cultivate a sense of community for vacationists, add navigation aids, and enhance the immersive experience. In addition, researchers have found that 360-degree VR videos could provide psychological resources and positive affective experiences, which allow individuals to effectively mitigate the adverse outcomes of mental health issues [81]. Thus, individuals who are in quarantine can use such VR products to fulfill their social gratification by connecting with other VR users, which is healthy for their psychological well-being during the pandemic.

Implications of this current research also offer guidance for improving users’ continued use of immersive videos during this or future unknown pandemics. Since the impacts of attitudes and VR engagement both played significant roles in users’ continued use of VR, practitioners should cultivate users’ subjective positive attitudes toward such immersive videos through intriguing, appealing, and interactive virtual content. Meanwhile, it is extremely critical for the VR industry to provide engaging experiences by designing VR platforms or content targeting specific groups of population and connecting closely to these users’ education, ethnicity, or socio-cultural background.

### 5.3. Limitations and Future Research

Although the results of this study have pertinent theoretical and practical contributions, several limitations must be mentioned. First, this current study was only conducted in the U.S. Since COVID-19 is a global health crisis, future studies could apply this proposed model to other cultures or countries to determine any potential cultural impact on the uses of and gratification achieved with VR technologies. For instance, future work might carry out analyses and comparisons using Bayesian networks between the U.S. and China. Second, this study only focused on the cognitive aspect of engagement. As engagement could include both emotional and cognitive dimensions, scholars could examine users’ emotional engagement and explore its impact on their attitudes and behaviors. The potential different relationships between gratifications and cognitive and emotional engagement deserve further exploration as well through experimental study. Last but not least, this current research only describes the different response data obtained for each gratification. In this sense, it would be interesting for future scholars to explore the reasons why participants use 360-degree VR videos in a certain way. Extending the current framework by exploring how demographic variables (e.g., age and gender), perceived trust, or risk might influence users’ gratifications and engagement in the context of VR campaigns might serve as a fruitful area for further research.

## 6. Conclusions

When the pandemic impacted global well-being, VR technology provided a new opportunity for users to experience reality and virtually reconnect with each other. Previous interdisciplinary research in the field of communication, management information system, computer science, and marketing has identified the importance of 360-degree VR videos. However, updated research on the motivations behind using such immersive videos within the context of post-pandemic is lacking. How to facilitate users’ engagement and continued use remains unknown. A major contribution of this study is to identify five types of gratification, systematically examine the reasons people use VR technologies, and test the different influences of gratifications on VR engagement in one theoretical framework. Furthermore, the results provide implications for global recovery and illustrate how to improve users’ engagement and continued use of immersive videos.

## Figures and Tables

**Table 1:** Gratifications studied in previous VR research.

Literature	VR Applications	Types of Gratifications				
		Utilitarian	Hedonic	Sensual	Social	Symbolic
Alzayat and Lee (2021) [28]	VR retail environment	√	√			
Herz and Rauschnabel (2019) [29]	VR glasses	√	√			
Kim and Lee (2021) [26]	360° VR art			√		√
Kim et al., (2020) [15]	VR tourism		√		√	
Wreford et al., (2019) [30]	VR event			√	√	
Zhang et al., (2019) [31]	Virtual try-on	√	√			
Ball et al., (2021) [32]	VR hardware				√	
Kim and Hall (2019) [33]	VR tourism		√			

**Table 2:** Inter-correlation between the constructs and the square root of AVEs (Fornell–Larcker criterion).

Variables	Navigation	Enjoyment	Realism	Community	Coolness	VR Engagement	User Attitude	Continued Use
Navigation	**0.86**							
Enjoyment	0.79 **	**0.85**						
Realism	0.80 **	0.73 **	**0.83**					
Community	0.81 **	0.70 **	0.77 **	**0.89**				
Coolness	0.72 **	0.74 **	0.72 **	0.67 **	**0.82**			
VR Engagement	0.74 **	0.74 **	0.72 **	0.72 **	0.65 **	**0.81**		
User Attitude	0.68 **	0.71 **	0.66 **	0.64 **	0.62 **	0.71 **	**0.91**	
Continued Use	0.79 **	0.78 **	0.77 **	0.74 **	0.72 **	0.78 **	0.74 **	**0.93**

** Correlation is significant at the 0.01 level (2-tailed). Values in the diagonal bolded are the square roots of AVE, and the off-diagonals are correlations. VR means virtual reality.

**Table 3:** Results of the measurement model.

Factor (Cronbach’s α)	Measurement Item	Factor Loadings ^a^	AVE/CR
Utilitarian gratification: Navigation (α = 0.90)	It allows me to link to other pieces of information.	0.87	AVE= 0.74
	It offers a number of visual aids for more effective use	0.88	CR = 0.90
	The interface helps me every step of the way	0.83	
Hedonic gratification: Enjoyment (α = 0.90)	It is fun to explore	0.85	AVE = 0.72
	It lets me play	0.82	CR = 0.88
	I enjoy escaping into a virtual world	0.87	
Sensual gratification: Realism (α = 0.88)	I know the content is real and not made up.	0.77	AVE = 0.69
	It is like communicating face-to-face.	0.88	CR = 0.87
	The VR experience is very much like real life.	0.89	
Social gratification: Community (α = 0.93)	I can connect with other followers.	0.88	AVE = 0.79
	It allows me to expand my social network.	0.88	CR = 0.92
	It makes me realize that I am part of a community.	0.90	
Symbolic gratification: Coolness (α = 0.87)	It is unique.	0.89	AVE = 0.68
	It is innovative.	0.81	CR = 0.86
	It is different.	0.77	
VR Engagement (α = 0.92)	I thought most about things having to do with the 360-degree VR video.	0.80	AVE = 0.66
	I imagined precisely what it must be like to further explore the world presented in the 360-degree VR video.	0.86	CR = 0.85
	I kept wondering whether the presentation in the 360-degree VR video could have personal meaning for me.	0.77	
User Attitude (α = 0.93)	The 360-degree VR video is appealing to me	0.92	AVE = 0.82
	The 360-degree VR video is attractive to me.	0.91	CR = 0.93
	The 360-degree VR video is interesting to me	0.88	
Continued Use (α = 0.93)	I would like to recommend others to watch 360-degree VR videos.	0.95	AVE = 0.87
	I will increase my use of 360-degree VR videos.	0.93	CR = 0.95
	Continuing to use 360-degree VR videos is something I would do in the future.	0.93	

Notes: ^a^ All factor loadings are significant at the level of *p* < 0.001.

## Data Availability

The data are not publicly available due to privacy or ethical restrictions.
